# Correction for Hirsch et al., “Complete genomes of two *Variovorax* endophytes isolated from surface-sterilized alfalfa nodules”

**DOI:** 10.1128/mra.01448-25

**Published:** 2026-02-04

**Authors:** Ann M. Hirsch, María del Pilar Martínez Hidalgo, Ethan Humm, Mila Rubbi, Giorgia Del Vecchio, Sung Min Ha, Matteo Pellegrini, Robert P. Gunsalus

**Affiliations:** 1 Department of Molecular, Cell, and Developmental Biology, University of California 5116, Los Angeles, California, USA; 2 Department of Biología y Geología, Física y Química Inorgánica, Universidad Rey Juan Carlos 253323 , Madrid, Spain; 3 Department of Microbiology, Immunology, and Molecular Genetics, University of California 5116, Los Angeles, California, USA; 4 Department of Integrative Biology and Physiology, University of California 5116, Los Angeles, California, USA; 5 UCLA DOE Institute, University of California 5116, Los Angeles, California, USA

## AUTHOR CORRECTION

Vol. 13, no. 8, e00336-24, 2024, https://doi.org/10.1128/mra.00336-24. The byline and affiliations should appear as given in this correction. María del Pilar Martínez Hidalgo was inadvertently omitted.

